# Moderately Low Magnesium Intake Impairs Growth of Lean Body Mass in Obese-Prone and Obese-Resistant Rats Fed a High-Energy Diet

**DOI:** 10.3390/nu8050253

**Published:** 2016-04-28

**Authors:** Jesse Bertinato, Christopher Lavergne, Sophia Rahimi, Hiba Rachid, Nina A. Vu, Louise J. Plouffe, Eleonora Swist

**Affiliations:** 1 Nutrition Research Division, Health Products and Food Branch, Health Canada, Sir Frederick G. Banting Research Centre, 251 Sir Frederick Banting Driveway, Ottawa, ON K1A 0K9, Canada; clave024@uottawa.ca (C.L.); sophia.rahimi@mail.mcgill.ca (S.R.); hiba-rachid-92@hotmail.com (H.R.); nvu008@uottawa.ca (N.A.V.); louise.j.plouffe@hc-sc.gc.ca (L.J.P.); eleonora.swist@hc-sc.gc.ca (E.S.); 2 Department of Biochemistry, Microbiology, and Immunology, University of Ottawa, Ottawa, ON K1H 8M5, Canada; 3 Department of Biology, University of Ottawa, Ottawa, ON K1N 6N5, Canada; 4 Food Science and Nutrition Program, Carleton University, Ottawa, ON K1S 5B6, Canada

**Keywords:** bone, diet, glucose, growth, lean body mass, magnesium deficiency, obesity, rat

## Abstract

The physical and biochemical changes resulting from moderately low magnesium (Mg) intake are not fully understood. Obesity and associated co-morbidities affect Mg metabolism and may exacerbate Mg deficiency and physiological effects. Male rats selectively bred for diet-induced obesity (OP, obese-prone) or resistance (OR, obese-resistant) were fed a high-fat, high-energy diet containing moderately low (LMg, 0.116 ± 0.001 g/kg) or normal (NMg, 0.516 ± 0.007 g/kg) Mg for 13 weeks. The growth, body composition, mineral homeostasis, bone development, and glucose metabolism of the rats were examined. OP and OR rats showed differences (*p* < 0.05) in many physical and biochemical measures regardless of diet. OP and OR rats fed the LMg diet had decreased body weight, lean body mass, decreased femoral size (width, weight, and volume), and serum Mg and potassium concentrations compared to rats fed the NMg diet. The LMg diet increased serum calcium (Ca) concentration in both rat strains with a concomitant decrease in serum parathyroid hormone concentration only in the OR strain. In the femur, Mg concentration was reduced, whereas concentrations of Ca and sodium were increased in both strains fed the LMg diet. Plasma glucose and insulin concentrations in an oral glucose tolerance test were similar in rats fed the LMg or NMg diets. These results show that a moderately low Mg diet impairs the growth of lean body mass and alters femoral geometry and mineral metabolism in OP and OR rats fed a high-energy diet.

## 1. Introduction

Magnesium (Mg) is an essential nutrient and co-factor in hundreds of metabolic reactions in the body. Mg is required for cell proliferation, cellular energy production, mineral metabolism, bone development, and glucose homeostasis [1,2,3,4]. Nutrition surveys in North America indicate that Mg consumption is below recommended intakes for a large segment of the population [5,6]. Furthermore, diseases such as type 2 diabetes [7] and use of certain medications [8] can increase Mg loss and predispose individuals to Mg deficiency. The low Mg intakes in comparison to current recommendations combined with the high prevalence of factors that can increase Mg requirements raise concern about widespread Mg deficiency. Biochemical data lend further support. Hypomagnesemia (low serum Mg) exists in the general population and the incidence is high in certain subpopulations [7,9,10].

Despite evidence suggesting Mg deficiency in the general population, the health implications are unclear. Several factors account for this, including concerns that dietary recommendations for Mg [11] may be set too high [12] and that much of the evidence relating lower Mg intake or serum Mg with diseases and health conditions is based on observational studies. In addition, overt symptoms of Mg deficiency (e.g., hypocalcemia) are rarely observed in the general population and there is uncertainty regarding the serum Mg concentration needed for optimal health.

Since Mg is required for many enzymatic reactions, Mg deficiency can presumably affect numerous physiological processes. Some studies have reported changes in body composition with dietary Mg restriction. In rats, maternal and postnatal feeding of a Mg-deficient diet decreased body weight, lean body mass, and fat free mass and increased percentage body fat in the offspring [13,14]. In contrast, body weight, fat mass, and lean mass were similar in rats fed a high-fat diet containing normal or low Mg beginning after weaning [15].

Studies in rats and mice have shown that dietary Mg restriction impairs bone growth, changes bone architecture, and increases bone fragility [16,17,18,19,20,21]. An uncoupling of bone formation and bone resorption was observed in rats fed a very low Mg diet [22]. Mg-depleted rats showed greater bone resorption with an increase in the number of tartrate-resistant acid phosphatase-positive osteoclasts and a decrease in the number of osteoblasts per area of bone surface [22]. With less severe dietary Mg restriction (diets containing 10%–50% of nutritional requirement), the number of osteoblasts per bone surface were not affected in the distal femur of rats [16,17,18,23]. However, rats fed a Mg-deficient diet containing as high as 50% of nutritional requirement showed a greater number of osteoclasts per bone surface, reduced bone mineral content, and lower percentage of trabecular bone volume in the distal femur [16].

The effects of Mg deficiency on parathyroid hormone (PTH) secretion/action and calcium (Ca) homeostasis may partly account for the observed effects on bone [23]. In humans, severe Mg deficiency impairs PTH secretion, causing hypocalcemia [24]. In rats, severe Mg deficiency increases serum Ca and decreases serum PTH [18,20]. With severe dietary Mg restriction, competition between Mg and Ca for intestinal absorption and reabsorption in the kidney may be diminished in rats, causing a rise in serum Ca concentration [23]. The observed decrease in serum PTH may be a consequence of the rise in serum Ca or direct impairment of PTH secretion [23]. Under conditions of milder dietary Mg restriction, a decrease in serum Ca and an increase in serum PTH have been reported in rats [16]. With less severe Mg restriction, competition between Mg and Ca may still exist and the small reduction in serum Mg may affect the Ca-sensing receptor in a similar way as Ca, thus increasing PTH secretion [23]. It is only when the degree of Mg deficiency becomes more pronounced that PTH secretion is reduced.

There is evidence to suggest that Mg deficiency may contribute to insulin resistance and glucose intolerance [13]. Maternal and perinatal Mg restriction in rats induced insulin resistance and decreased insulin response to a glucose challenge [13]. A recent study showed decreased phosphorylation of proteins involved in the insulin-signaling pathway in rats fed a Mg-deficient, high-fat diet, suggesting decreased insulin sensitivity, but blood glucose and insulin concentrations did not differ compared to rats fed a normal Mg diet [15]. In human studies, lower Mg intakes or serum Mg concentrations have been associated with reduced bone mineral density [25], type 2 diabetes [26,27,28,29], and poorer insulin sensitivity and glucose control [9,30].

At present the physiological effects of moderately low Mg intakes are incompletely understood. The objective of this study was to examine physical and biochemical changes in rats fed moderately low Mg in the context of a high-fat, high-energy diet. Effects on body weight, body composition, mineral metabolism, bone development, and glucose homeostasis were investigated. Given that obesity and associated co-morbidities (e.g., type 2 diabetes) can affect Mg metabolism, effects were examined in rat strains selectively bred for susceptibility or resistance to diet-induced obesity. We report that a moderate decrease in dietary Mg impairs growth of lean body mass, alters femoral geometry, and induces changes in serum and bone mineral concentrations in these rat strains.

## 2. Materials and Methods

### 2.1. Animal Protocol and Diets

Fifty Crl:OP (CD) (OP, obese-prone) and 50 Crl:OR (CD) (OR, obese-resistant) male rats (Charles River Canada, St. Constant, QC, Canada) of six weeks of age were used in this study. OP (*n* = 25/diet group) and OR (*n* = 25/diet group) rats were fed high-fat, high-energy diets (Dyets, Inc., Bethlehem, PA, USA) containing moderately low (LMg, 0.116 ± 0.001 g/kg diet) or normal (NMg, 0.516 ± 0.007 g/kg diet) Mg. Compositions and energy densities of the diets are shown in Table S1. Diets were formulated using the AIN-93G mineral mix [31] without Mg and supplemented with either 0.100 (LMg) or 0.500 (NMg) g of Mg (as Mg oxide) per kg diet. Mg concentrations in the final diets were slightly higher than expected based on the amount of Mg oxide added. The additional Mg comes from other diet ingredients. Diets were a modified version of the Research Diets, Inc. D12266B formulation used previously to induce obesity in rats [32,33,34]. Diets were pelleted for more accurate measurement of food consumption.

OP and OR rats were assigned to diet groups based on initial body weight so that mean body weights in each diet group were similar at the start of the study. Rats were housed individually in solid-bottom cages held in vented racks. Cages had a wire-grille insert on the bottom and contained a stainless steel platform and shelter. Rats were put on a 12:12-h light-dark cycle and had free access to food and demineralized water throughout the study. Food consumption and body weight for each rat was measured twice a week. Food consumption was determined by measuring food missing from the feeder. Results for body weight measurements and parameters related to food consumption were not reported between days 46–64 and 78–81 because some rats were fasted on the selected days of measurements. The body composition of each rat was measured at the beginning (week 0), middle (weeks 8 and 9), and end (week 14) of the study using an EchoMRI-4in1™ system (EchoMRI, Houston, TX, USA).

After 13 weeks of feeding the diets, rats were fasted overnight (~12 h) in metabolic cages for collection of urine. Rats were then killed by exsanguination under general isoflurane anesthesia. Blood was collected from the abdominal aorta by syringe and dispensed into a Trace Element Serum tube (14-816-154, Thermo Fisher Scientific, Ottawa, ON, Canada) for isolation of serum. Hind legs and adipose depots were extracted and immediately frozen on dry ice and then stored at −80 °C until analysis. The experimental protocol was approved by the Health Products and Food Branch Animal Care Committee of Health Canada (Protocol No.: 2014-007).

During the study, five OR rats fed the LMg diet and two OR rats fed the NMg diet died unexpectedly without symptoms. The number of deaths did not differ (*p* ≥ 0.05, Fisher’s exact test) among diet groups. Serology reports from sentinel rats were negative, suggesting that an infectious agent was not responsible. Post-mortem examination did not reveal significant lesions suggestive of infection, toxicity, or lymphoma and the final judgment was sudden death of undetermined cause. Results from an additional four rats were excluded from the analyses. These rats were euthanized following unintentional gavage of solution into the lungs during a practice (*n* = 3) and the experimental (*n* = 1) oral glucose tolerance test (OGTT).

### 2.2. OGTT

During week 13 of the study an OGTT was performed on subgroups (*n* = 10/group) of OP and OR rats with the highest and lowest percentage body fat measurements at weeks 8–9 of the study, respectively. Following an overnight fast (~12 h), a 0.4 g/mL dextrose solution was orally administered to the rats by gavage (2 g dextrose/kg body weight). Blood samples (~250 μL) were drawn from the tail vein before dextrose dosing (0 min) and 30, 60, and 120 min after dosing. Blood samples were dispensed into BD Microtainer™ tubes with lithium heparin (13-680-62, Thermo Fisher Scientific) for isolation of plasma. Plasma samples were immediately frozen at −80 °C until analysis.

### 2.3. Femur Isolation and Physical Measurements

Femurs were isolated from the right leg. Skin and flesh was removed using a scalpel and forceps. Wet and dry femur weights were measured using a Mettler Toledo AT261 delta range analytical balance (Mettler Toledo, Mississauga, ON, Canada). Femur length and width were measured using a dial caliper (Mitutoyo Canada Inc., Toronto, ON, Canada). Femur length corresponds to the distance from the greater trochanter to the lateral condyle. Femur width was the distance between medial and lateral surfaces at midshaft. Femur volume was determined using a PYREX™ specific gravity bottle (01-716, Thermo Fisher Scientific). Femur volume was calculated as the mass of water displaced by the femur in grams divided by the density of water (0.99997 g/cm^3^). Femur density was calculated as the wet weight in grams divided by the volume in cubic centimeters.

### 2.4. Mineral Analyses

Diets (~1 g samples) and femurs were placed in preweighted quartz beakers and dried overnight at 100 °C in an Isotemp^®^ oven (Thermo Fisher Scientific). Samples were cooled, placed in a desiccator for 1 h and then weighed to obtain dry weights. Diets and femurs were ashed using a combination of dry ashing using an Isotemp^®^ Programmable Forced Draft Furnace (Thermo Fisher Scientific) and wet ashing using concentrated trace metal grade nitric acid (Thermo Fisher Scientific). Ashes were solubilized in dilute nitric acid. Solubilized ashes and urine samples were analyzed for mineral concentrations using a 700 Series inductively coupled plasma optical emission spectrometer (Agilent Technologies Canada Inc., Mississauga, ON, Canada). Operating conditions have been described previously [35]. Concentrations of minerals were derived from a standard calibration curve prepared using the CALEDON-88 multi-element standard (Inorganic Ventures, Christiansburg, VA, USA). Analytical accuracy was verified using National Institute of Standards and Technology traceable reference material™ (SCP Science, Baie D’Urfé, QC, Canada).

### 2.5. Assays

Mineral concentrations in serum and urine creatinine were measured using the ABX Pentra 400 chemistry analyzer (HORIBA Instruments Inc., Irvine, CA, USA). Plasma insulin and glucose were measure using the Rat Ultrasensitive Insulin ELISA (80-INSRTU-E01, Alpco Diagnostics, Salem, NH, USA) and Glucose Colorimetric Assay Kit (10009582, Cayman Chemical, Ann Arbor, MI, USA), respectively. Serum PTH and osteocalcin were measured using the Rat BioActive Intact PTH ELISA Kit (60-2700, Immutopics, Inc., San Clemente, CA, USA) and Osteocalcin Rat Enzyme Immunoassay Kit (BT-490, Alfa Aesar, Ward Hill, MA, USA), respectively. Urine deoxypyridinoline (DPD) was determined using the MicroVue™ DPD EIA Kit (8007, Quidel Corporation^®^, San Diego, CA, USA).

### 2.6. Statistical Analyses

Results are reported as means ± SD. Two-way ANOVA was used for analysis of parameters measured at a single time point to examine the overall effects of strain and diet. Fisher’s least significant difference *post hoc* test was performed for parameters with a significant (*p* < 0.05) strain × diet interaction. Mixed-design ANOVA was used for analysis of parameters measured at multiple time points to determine the effects and interactions of time, strain, and diet. For parameters with a significant time × strain or time × diet interaction, univariate results are presented for the effect of strain or diet at each time point, respectively. Homogeneity of variances was assessed using Levene’s test. Data that showed unequal variances were transformed prior to analysis. Fisher’s exact test was used to determine differences in proportions. The area under the glucose and insulin curves was calculated using the trapezoidal rule with the Area below Curves function in SigmaPlot 12.5 (Systat Software Inc., Chicago, IL, USA). Statistical analyses were performed using Statistica 7 (StatSoft, Tulsa, OK, USA). Statistical significance was set at *p* < 0.05.

## 3. Results

**Figure 1:**
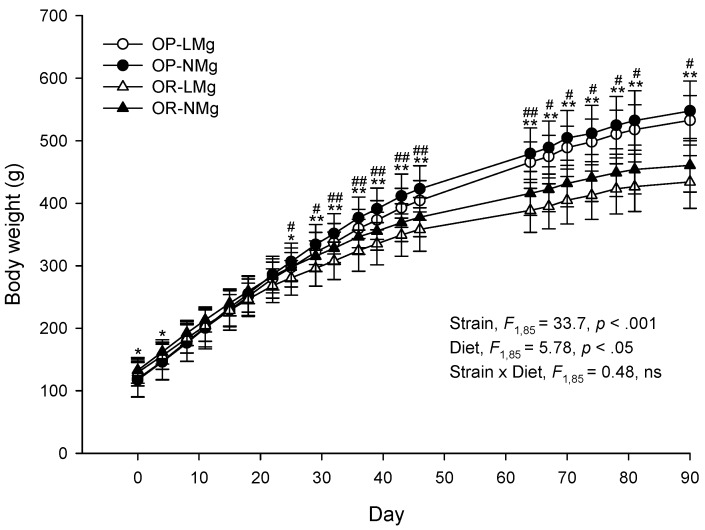
Body weights of rats. Results are presented as means ± SD, *n* = 18–25. Results were analyzed by mixed-design ANOVA to determine effects and interactions of time, strain, and diet. Time × strain and time × diet interactions (*p* < 0.05) were observed and univariate results are shown for effects of strain (*, *p* < 0.05; **, *p* < 0.001) and diet (^#^, *p* < 0.05; ^##^, *p* < 0.01). ns, *p* ≥ 0.05.

This study examined the effects of a moderately low Mg diet high in fat and energy on growth, body composition, energy intake, energy efficiency, mineral homeostasis, bone development, and glucose metabolism in OP and OR rats. The effect of rat strain and diet on each parameter is presented. Body weights differed between rat strains and between rats fed the low or normal Mg diets (Figure 1). Body weights at the start of the study were higher for OR than OP rats. From day 25 to the end of the study, OP rats were heavier than OR rats and rats fed the low Mg diet were lighter than rats fed the normal Mg diet.

Percentage lean body mass was higher, but total lean body mass was lower in OR compared to OP rats at weeks 8–9 and week 14 (Table 1). Rats fed the low Mg diet had lower total lean body mass at weeks 8–9 and week 14. Percentage body fat, total body fat, and weight of four distinct fat depots were higher in OP compared to OR rats at week 14 (Table 1). Percentage and total body fat did not differ between rats fed the low or normal Mg diets. Weight of mesenteric fat was lower in rats fed the low Mg diet.

**Figure 2:**
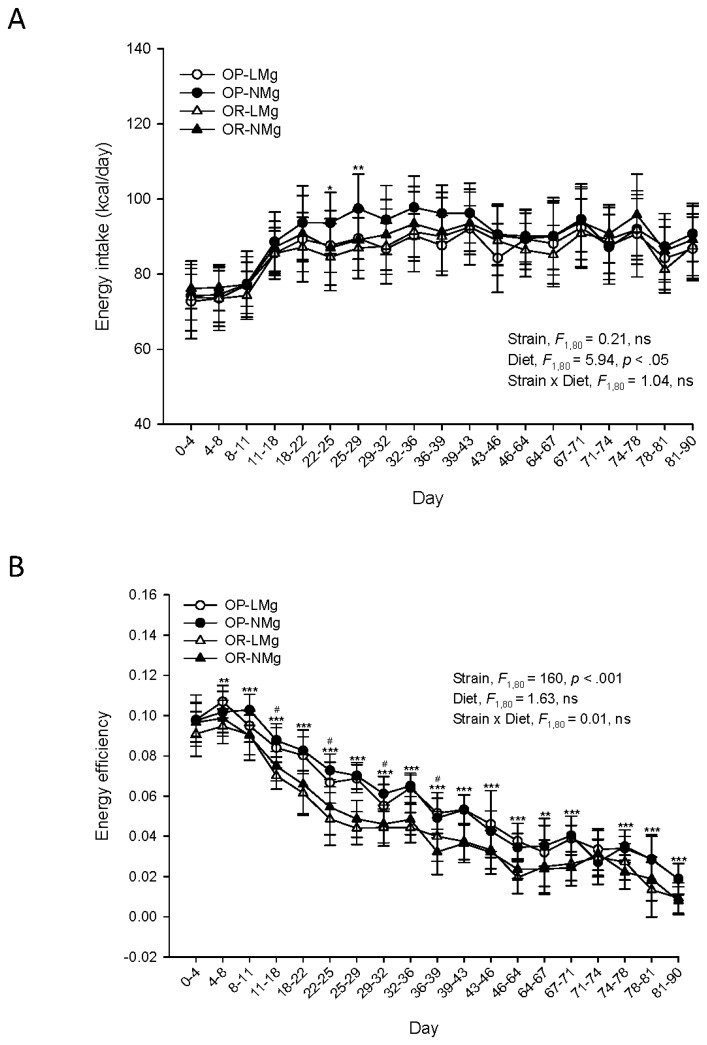
Energy intake and energy efficiency of rats. Results are presented as means ± SD, *n* = 17–25. Results for energy intake (**A**); and energy efficiency (**B**) were analyzed by mixed-design ANOVA to determine effects and interactions of time, strain, and diet. Univariate results are shown for effects of strain (*, *p* < 0.05; **, *p* < 0.01; ***, *p* < 0.001) or diet (^#^, *p* < 0.05) when a significant (*p* < 0.05) time × strain or time × diet interaction was observed, respectively. Energy efficiency = (body weight gain (g/day)/energy intake (kcal/day)). ns, *p* ≥ 0.05.

Energy intakes did not differ between rat strains (Figure 2A). Rats fed the low Mg diet had lower energy intakes compared to rats fed the normal Mg diet. OP rats had superior energy efficiency compared to OR rats (Figure 2B). Energy efficiency was similar for rats fed the low or normal Mg diets. Food consumption did not differ between rat strains, but was lower for rats fed the low Mg diet (Figure S1). Similarly, Mg intake was comparable between rat strains, but was lower for rats fed the low Mg diet (Figure S2).

Concentrations of minerals and markers of bone metabolism were examined in the serum (Table 2) and urine (Table 3) of the rats. Concentrations of minerals and bone markers in serum and urine differed between rat strains. Serum and urine Mg concentrations were lower in rats fed the low Mg diet. Serum Ca concentrations were higher and serum potassium (K) concentrations were lower in rats fed the low Mg diet. The low Mg diet decreased serum PTH concentration only in OR rats (Table 2). Serum osteocalcin and urine DPD concentrations were similar in rats fed the low or normal Mg diets (Table 2 and Table 3).

Femoral mineral concentrations and physical measurements were assessed in the rats. OP and OR rats showed differences in Mg, K, and sodium (Na) concentrations in femur (Table 4). Concentration of Mg was lower and concentrations of Ca and Na were higher in femurs of rats fed the low Mg diet.

Measurements for femur width, dry weight, and density were higher, whereas femur length:width ratio was lower in OP compared to OR rats (Table 5). Measurements for femur width, wet weight, dry weight, and volume were lower in rats fed the low Mg diet. The low Mg diet increased femur length:width ratio only in OR rats.

**Figure 3:**
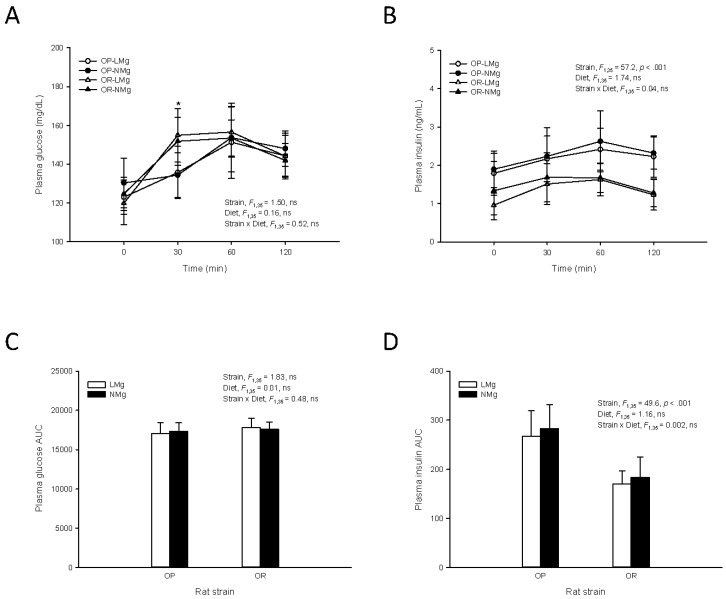
Plasma glucose and insulin during an oral glucose tolerance test conducted at week 13 of the study. Plasma glucose and insulin concentrations (**A**,**B**) and the respective area under the curve (AUC) (**C**,**D**); Dextrose solution (0.4 g/mL) was orally administered to the rats (2 g dextrose/kg body weight) after an overnight fast. Blood was collected from the tail vein before dosing (0 min) and 30, 60, and 120 min after dosing. Results are displayed as means ± SD, *n* = 9–10. Results were analyzed by mixed-design ANOVA to determine the effects and interactions of time, strain, and diet (**A**,**B**); For glucose a significant (*p* < 0.05) time × strain interaction was observed and univariate results are shown for effect of strain (*, *p* < 0.001). AUC results were analyzed by two-way ANOVA (**C**,**D**). ns, *p* ≥ 0.05.

An OGTT was performed on a subgroup of rats in each group to investigate the effect of the low Mg diet on glucose homeostasis. Mean percentage body fat for these rats at the end of the study were 25.9% ± 2.5% (OP-LMg), 26.6% ± 2.1% (OP-NMg), 16.7% ± 1.4% (OR-LMg), and 17.3% ± 1.6% (OR-NMg). At the 30 min time point, plasma glucose concentration was higher in OR rats compared to OP rats regardless of diet (Figure 3A). Plasma glucose concentrations were similar for OP and OR rats fed the low or normal Mg diets at each time point (Figure 3A). The area under the glucose curve did not differ between rat strains or rats fed the low or normal Mg diets (Figure 3C). OP rats had higher plasma insulin concentrations at each time point compared to OR rats (Figure 3B). Area under the insulin curve was also greater for OP rats (Figure 3D). Plasma insulin concentrations at each time point and area under the insulin curve were similar in OP and OR rats fed the low or normal Mg diets.

## 4. Discussion

This study examined physical and biochemical changes in rats fed a moderately low Mg diet with the aim of identifying physiological processes sensitive to reduction in Mg intake. Obesity and associated co-morbidities such as type 2 diabetes affect Mg metabolism [7] and may increase vulnerability to Mg deficiency and adverse health outcomes. For this reason effects were investigated in rats selectively bred for susceptibility or resistance to diet-induced obesity. The high-fat, high-energy diets used in this study induced differences in adiposity between OP and OR rats validating our experimental model. OP rats had greater fat mass and percentage body fat at weeks 8–9 and at the end of the study.

The low Mg diet contained 23% of nutritional requirement for growing rats [31]. This degree of deficiency may exist in some individuals in the extreme lower percentiles of usual Mg intakes [11] (pp. 392–393). The low Mg diet depressed Mg status in both rat strains as evidenced by reductions in Mg concentrations in serum, urine, and femur.

Some evidence suggests that Mg status may affect body composition. In a placebo-controlled, randomized trial, supplementation of overweight women with 250 mg of Mg daily for eight weeks resulted in an increase in lean body mass and decrease in fat mass compared to baseline values [36]. In rats, maternal and postnatal Mg restriction caused a decrease in lean body mass and increase in percentage body fat in the offspring [13,14]. In the present study the low Mg diet decreased body weight and lean body mass in both rat strains, demonstrating that dietary Mg restriction after weaning impairs the growth of lean body mass. The low Mg diet did not affect percentage or total fat mass, indicating a greater effect on growth of lean mass as opposed to accumulation of fat. Energy efficiency of rats fed the low or normal Mg diets was similar, meaning that rats consumed a similar amount of energy from food for a similar amount of body weight gain. These results indicate that metabolic inefficiency was not the cause of the lower body weight and lean mass. The data also do not support a repartitioning of dietary energy from lean body mass to adipose tissue in rats fed low Mg since fat mass did not increase. The lower energy intake (and food consumption) observed for rats fed the low Mg diet is likely explained by the impaired growth and smaller size of the rats. Together, these results demonstrate that growth of lean body mass is sensitive to decreases in Mg intake and a moderately low Mg diet does not promote adiposity in rats selectively bred for susceptibility or resistance to diet-induced obesity.

There is compelling evidence indicating that Mg plays an important role in the control of cell proliferation [3,4] and protein synthesis [37]. Impaired cell growth and/or downregulation of protein synthesis may have contributed to the observed decrease in growth of lean body mass of rats fed the low Mg diet. Effects on the secretion or action of anabolic hormones such as insulin-like growth factor 1 or testosterone could also explain the impaired growth of lean body mass [38,39].

The low Mg diet reduced femur size (width, weight, and volume) in both rat strains and increased femoral length:width ratio in OR rats. These findings are noteworthy since the physical characteristics of a bone (shape and size) influence its mechanical strength [40]. Femoral structural geometry has been shown to adapt to mechanical loading [41], and therefore these femoral changes may be a consequence of a lesser mechanical load because of the lower body weight of rats fed the low Mg diet. Even though bone density and markers of bone formation (serum osteocalcin) and resorption (urine DPD:creatinine ratio) were unaffected by diet, specific effects on processes that influence bone development cannot be excluded. Bone development in rats is sensitive to reduction in Mg intake [16,17,18,19]. A low Mg diet containing 50% of nutritional requirement decreased the percentage of trabecular bone volume and bone mineral content of the distal femur in rats [16]. It has been proposed that increased release of substance P and inflammatory cytokines in response to Mg depletion may contribute to bone loss [16,17]. The increase in femur Ca and Na concentrations observed in this study for rats fed the low Mg diet may be a result of the lower amount of Mg incorporated into the bone.

Serum Ca has been shown to increase in rats and mice in response to moderate or severe Mg deficiency [18,20,21]. Serum Ca increased in both rat strains fed the low Mg diet. In OR rats there was an associated decrease in serum PTH, which excludes hyperactivity of the parathyroid gland as the cause. Ca and Mg compete for intestinal absorption and reabsorption in the kidney [42,43]. Increased Ca absorption and/or reabsorption resulting from diminished Mg antagonism may account for the observed rise in serum Ca. The associated decrease in PTH in OR rats may be explained by hypoactivity of the parathyroid gland in response to the rise in serum Ca. This is supported by experiments in Mg-deficient rats demonstrating increased blood Ca and hypoactivity of the parathyroid gland determined by histologic and morphometric analyses [20]. OP rats fed the low Mg diet did not show a reduction in PTH despite a rise in serum Ca. The higher basal serum Ca (and perhaps PTH) in OR rats may have sensitized the parathyroid gland to an additional rise in serum Ca.

A decrease in serum K concentration was observed for rats fed the low Mg diet. A reduction in serum K is often associated with Mg deficiency [44]. It has been proposed that Mg deficiency causes K wasting by increasing renal K excretion [44]. In this study, urine K concentration was not elevated in rats fed low Mg; however, urine minerals were only measured at the end of the study and thus we cannot comment on urinary excretion of minerals at earlier time points in the study.

Lower Mg intakes and serum Mg concentrations have been associated with type 2 diabetes [26,27,28,29]. A number of mechanisms have been proposed to explain the negative effect of diabetes on Mg status including increased renal Mg loss from glycosuria [7]. Whether moderately low Mg intakes adversely affect glucose homeostasis is less clear. In this study, the effects of the low Mg diet on plasma insulin and glucose concentrations were examined during an OGTT. OP rats with the highest percentage body fat and OR rats with the lowest percentage body fat were selected to allow for investigation of any modifying effect of adiposity. Plasma insulin concentrations were higher in OP than OR rats, which is in agreement with the greater insulin resistance previously described for obese-prone rats [45,46,47]. Plasma glucose and insulin concentrations were unaffected by diet, indicating that any effects of the low Mg diet on glucose control and insulin resistance were minor. The results also indicate that the differences in adiposity and insulin resistance between OP and OR rats did not influence the outcome. At the 30 min time point, OR rats showed higher plasma glucose compared to OP rats, irrespective of diet. This may be explained by slower intestinal glucose absorption and reduced entry of glucose into the bloodstream in OP rats. Delayed and lower glucose absorption has been reported for obese compared to lean rats [48]. The results of this study differ from the results of an earlier study that showed greater insulin resistance, impaired glucose tolerance, and lower insulin response to a glucose challenge in Mg-restricted rats [13]. An important distinction, however, is that rats in that study were born to dams that were deprived of Mg before conception and during pregnancy.

OP and OR rats showed differences in body composition, femoral physical measurements, markers of bone metabolism, plasma insulin, and serum and femur mineral concentrations. Despite these differences, for most parameters examined, an interaction between diet and strain was not observed, indicating that the low Mg diet had a similar effect in both rat strains. The exceptions were a decrease in serum PTH concentration and an increase in the femur length:width ratio, which was only observed in OR rats fed the low Mg diet. These results indicate that the greater adiposity in OP rats did not exacerbate the physical and biochemical changes induced by the low Mg diet.

The results from this study should be interpreted in the context of the experimental design. Rats were fed low Mg in the background of a high-fat, high-energy diet and therefore the effects may not be generalizable to lower fat and energy diets. It should be noted, however, that the low and normal Mg diets only differed in Mg content and therefore the reported physical and biochemical differences can be attributed to differences in Mg intake. It should also be mentioned that rats in this study were selectively bred for susceptibility or resistance to diet-induced obesity. Peculiarities in genetic makeup may have predisposed these rats to the effects of a low Mg diet. Caution is also warranted when extending these findings to humans, in particular effects on the Ca–PTH axis. Rats develop hypercalcemia in response to a moderate Mg deficiency, whereas humans develop hypocalcemia secondary to hypoparathyroidism and reduced circulating PTH concentration [24,49,50,51].

## 5. Conclusions

This study has shown that moderately low Mg intake when consuming a high-fat, high-energy diet causes prominent physiological changes in both OP and OR rats. The low Mg diet reduced body weight gain and growth of lean body mass and bone (femur) of the rats. In addition, serum and bone mineral concentrations were altered. These results underscore the importance of evaluating these physiological parameters in future studies exploring the public health risks from low Mg consumption.

## Figures and Tables

**Table 1:** Body composition of rats.

Parameter	Groups				ANOVA		
	OP-LMg (*n* = 25)	OP-NMg (*n* = 23)	OR-LMg (*n* = 18)	OR-NMg (*n* = 23)	Strain	Diet	Strain × Diet
Lean (%) ^1^					*F*_1,85_ = 9.4, *p* < 0.01	*F*_1,85_ = 0.9, ns	*F*_1,85_ = 0.0, ns
wk 0	85.5 ± 1.8	85.4 ± 1.4	85.2 ± 1.5	85.3 ± 1.7	*F*_1,85_ = 0.3, ns		
wk 8–9	77.2 ± 3.4	76.1 ± 3.6	79.0 ± 2.3	78.2 ± 2.7	*F*_1,85_ = 8.68, *p* < 0.01		
wk 14	72.2 ± 3.5	72.1 ± 4.2	75.2 ± 2.5	74.5 ± 2.8	*F*_1,85_ = 13.5, *p* < 0.001		
Lean (g) ^1^					*F*_1,85_ = 34.7, *p* < 0.001	*F*_1,85_ = 4.19, *p* < 0.05	*F*_1,85_ = 0.32, ns
wk 0	83.9 ± 22.1	83.4 ± 23.3	92.1 ± 18.4	95.1 ± 17.3	*F*_1,85_ = 5.11, *p* < 0.05	*F*_1,85_ = 0.085, ns	
wk 8–9	333 ± 24	344 ± 33	297 ± 28	312 ± 21	*F*_1,85_ = 35.1, *p* < 0.001	*F*_1,85_ = 5.29, *p* < 0.05	
wk 14	387 ± 29	396 ± 29	331 ± 26	346 ± 21	*F*_1,85_ = 89.3, *p* < 0.001	*F*_1,85_ = 4.83, *p* < 0.05	
Fat (%) ^1^					*F*_1,85_ = 23.8, *p* < 0.001	*F*_1,85_ = 1.36, ns	*F*_1,85_ = 0.061, ns
wk 0	10.6 ± 1.1	10.6 ± 1.2	10.0 ± 1.2	10.1 ± 0.8	*F*_1,85_ = 4.74, *p* < 0.05		
wk 8–9	17.9 ± 3.3	18.9 ± 3.3	14.9 ± 2.2	15.9 ± 2.6	*F*_1,85_ = 22.6, *p* < 0.001		
wk 14	22.6 ± 3.4	22.9 ± 4.2	18.6 ± 2.7	19.7 ± 2.9	*F*_1,85_ = 25.0, *p* < 0.001		
Fat (g) ^1^					*F*_1,85_ = 44.5, *p* < 0.001	*F*_1,85_ = 3.24, ns	*F*_1,85_ = 0.079, ns
wk 0	10.4 ± 3.1	10.3 ± 3.0	10.9 ± 2.7	11.4 ± 2.7	*F*_1,85_ = 1.42, ns		
wk 8–9	78.0 ± 19.7	86.2 ± 19.6	56.6 ± 12.2	64.3 ± 15.0	*F*_1,85_ = 34.8, *p* < 0.001		
wk 14	122 ± 24	127 ± 30	83 ± 18	93 ± 21	*F*_1,85_ = 52.6, *p* < 0.001		
Ing fat (g) ^2^	11.2 ± 2.5	11.8 ± 3.2	6.4 ± 1.5	7.3 ± 2.0	*F*_1,85_ = 85.3, *p* < 0.001	*F*_1,85_ = 2.04, ns	*F*_1,85_ = 0.566, ns
Retro fat (g) ^2^	21.7 ± 3.7	22.2 ± 4.7	13.9 ± 3.2	15.3 ± 3.7	*F*_1,85_ = 77.3, *p* < 0.001	*F*_1,85_ = 1.48, ns	*F*_1,85_ = 0.259, ns
Mes fat (g) ^2^	10.0 ± 2.5	10.7 ± 2.8	6.0 ± 1.4	7.0 ± 1.6	*F*_1,85_ = 70.4, *p* < 0.001	*F*_1,85_ = 4.29, *p* < 0.05	*F*_1,85_ = 0.666, ns
Epi fat (g) ^2^	15.8 ± 2.9	15.9 ± 4.1	9.2 ± 2.7	10.9 ± 2.6	*F*_1,85_ = 74.1, *p* < 0.001	*F*_1,85_ = 2.02, ns	*F*_1,85_ = 1.37, ns

Values are means ± SD; ^1^ Analyzed by mixed-design ANOVA to determine the effects and interactions of time, strain, and diet. Univariate results are shown for each time point for parameters with significant (*p* < 0.05) time × strain or time × diet interaction; ^2^ Analyzed by two-way ANOVA; ns, *p* ≥ 0.05. Epi, epididymal; Ing, inguinal; Mes, mesenteric; Retro, retroperitoneal; wk, week.

**Table 2:** Serum minerals and bone markers.

Parameter	Groups				ANOVA ^1^		
	OP-LMg (*n* = 25)	OP-NMg (*n* = 23)	OR-LMg (*n* = 18)	OR-NMg (*n* = 23)	Strain	Diet	Strain × Diet
Mg (mmol/L)	0.52 ± 0.11	0.74 ± 0.12	0.63 ± 0.10	0.85 ± 0.13	*F*_1,85_ = 20.0, *p* < 0.001	*F*_1,85_ = 75.0, *p* < 0.001	*F*_1,85_ = 0.017, ns
Ca (mmol/L)	2.67 ± 0.10	2.58 ± 0.11	2.78 ± 0.11	2.70 ± 0.12	*F*_1,85_ = 23.2, *p* < 0.001	*F*_1,85_ = 12.4, *p* < 0.001	*F*_1,85_ = 0.090, ns
P (mmol/L)	1.77 ± 0.24	1.83 ± 0.22	1.68 ± 0.17	1.75 ± 0.12	*F*_1,85_ = 4.05, *p* < 0.05	*F*_1,85_ = 2.84, ns	*F*_1,85_ = 0.007, ns
K (mmol/L)	3.99 ± 0.33	4.20 ± 0.25	4.32 ± 0.31	4.44 ± 0.21	*F*_1,85_ = 23.1, *p* < 0.001	*F*_1,85_ = 7.43, *p* < 0.01	*F*_1,85_ = 0.54, ns
Na (mmol/L)	143 ± 1	143 ± 1	143 ± 1	143 ± 2	*F*_1,85_ = 0.6, ns	*F*_1,85_ = 0.8, ns	*F*_1,85_ = 0.9, ns
PTH (ng/L)	152 ± 75 ^c^	125 ± 34 ^c^	221 ± 81 ^b^	306 ± 165 ^a^	*F*_1,85_ = 48.2, *p* < 0.001	*F*_1,85_ = 0.89, ns	*F*_1,85_ = 5.30, *p* < 0.05
OC (μg/L)	25.6 ± 8.2	24.8 ± 6.5	19.9 ± 6.5	21.3 ± 4.0	*F*_1,85_ = 11.1, *p* < 0.01	*F*_1,85_ = 0.042, ns	*F*_1,85_ = 0.628, ns

Values are means ± SD; ^1^ Analyzed by two-way ANOVA. Fisher’s least significant difference *post hoc* test was performed for the parameter with a significant (*p* < 0.05) strain × diet interaction. Values within the row without a common superscript letter differ, *p* < 0.05. ns, *p* ≥ 0.05. Ca, calcium; K, potassium; Mg, magnesium; Na, sodium; OC, osteocalcin; P, phosphorus; PTH, parathyroid hormone.

**Table 3:** Urine minerals and deoxypyridinoline.

Parameter	Groups				ANOVA ^1^		
	OP-LMg (*n* = 25)	OP-NMg (*n* = 23)	OR-LMg (*n* = 18)	OR-NMg (*n* = 23)	Strain	Diet	Strain × Diet
Mg (mg/g Cr)	27 ± 10	92 ± 25	26 ± 11	100 ± 31	*F*_1,85_ = 0.01, ns	*F*_1,85_ = 308, *p* < 0.001	*F*_1,85_ = 0.84, ns
Ca (mg/g Cr)	14 ± 2	14 ± 2	17 ± 3	16 ± 3	*F*_1,85_ = 26.8, *p* < 0.001	*F*_1,85_ = 0.348, ns	*F*_1,85_ = 0.058, ns
P (mg/g Cr)	885 ± 302	960 ± 415	1323 ± 276	1261 ± 252	*F*_1,85_ = 29.4, *p* < 0.001	*F*_1,85_ = 0.009, ns	*F*_1,85_ = 1.01, ns
K (mg/g Cr)	2160 ± 430	2190 ± 610	2530 ± 550	2560 ± 550	*F*_1,85_ = 10.7, *p* < 0.01	*F*_1,85_ = 0.076, ns	*F*_1,85_ = 0.00, ns
Na (mg/g Cr)	367 ± 199	371 ± 171	198 ± 99	227 ± 110	*F*_1,85_ = 22.4, *p* < 0.001	*F*_1,85_ = 0.244, ns	*F*_1,85_ = 0.148, ns
DPD (nmol/mmol Cr)	93 ± 21	103 ± 24	159 ± 36	171 ± 49	*F*_1,85_ = 85.9, *p* < 0.001	*F*_1,85_ = 2.35, ns	*F*_1,85_ = 0.037, ns

Values are means ± SD; ^1^ Analyzed by two-way ANOVA; ns, *p* ≥ 0.05; Ca, calcium; Cr, creatinine; DPD, deoxypyridinoline; K, potassium; Mg, magnesium; Na, sodium; P, phosphorus.

**Table 4:** Concentrations of minerals in femur.

Parameter	Groups				ANOVA ^1^		
	OP-LMg (*n* = 25)	OP-NMg (*n* = 22)	OR-LMg (*n* = 18)	OR-NMg (*n* = 23)	Strain	Diet	Strain × Diet
Mg (mg/g DW)	2.88 ± 0.26	4.55 ± 0.15	3.61 ± 0.25	4.94 ± 0.15	*F*_1,84_ = 164, *p* < 0.001	*F*_1,84_ = 1266, *p* < 0.001	*F*_1,84_ = 3.50, ns
Ca (mg/g DW)	282 ± 8	279 ± 5	283 ± 11	275 ± 9	*F*_1,84_ = 0.71, ns	*F*_1,84_ = 9.77, *p* < 0.01	*F*_1,84_ = 2.08, ns
P (mg/g DW)	137 ± 4	139 ± 4	138 ± 5	136 ± 5	*F*_1,84_ = 0.53, ns	*F*_1,84_ = 0.03, ns	*F*_1,84_ = 2.36, ns
K (mg/g DW)	1.91 ± 0.11	1.96 ± 0.11	2.18 ± 0.17	2.17 ± 0.09	*F*_1,84_ = 86.8, *p* < 0.001	*F*_1,84_ = 0.45, ns	*F*_1,84_ = 1.33, ns
Na (mg/g DW)	9.18 ± 0.29	8.75 ± 0.21	8.94 ± 0.45	8.50 ± 0.22	*F*_1,84_ = 14.9, *p* < 0.001	*F*_1,84_ = 45.6, *p* < 0.001	*F*_1,84_ = 0.00, ns

Values are means ± SD; ^1^ Analyzed by two-way ANOVA; ns, *p* ≥ 0.05; Ca, calcium; DW, dry weight; K, potassium; Mg, magnesium; Na, sodium; P, phosphorus.

**Table 5:** Physical measurements of femur.

Parameter	Groups				ANOVA ^1^		
	OP-LMg (*n* = 25)	OP-NMg (*n* = 23)	OR-LMg (*n* =1 8)	OR-NMg (*n* = 23)	Strain	Diet	Strain × Diet
Length (mm) ^2^	38.8 ± 0.6	39.0 ± 1.0 ^4^	38.8 ± 0.7	39.1 ± 0.6	*F*_1,84_ = 0.0, ns	*F*_1,84_ = 2.4, ns	*F*_1,84_ = 0.1, ns
Width (mm) ^3^	4.59 ± 0.12	4.63 ± 0.28	4.16 ± 0.21	4.32 ± 0.17	*F*_1,85_ = 76.2, *p* < 0.001	*F*_1,85_ = 5.65, *p* < 0.05	*F*_1,85_ = 2.78, ns
Length:width ratio	8.46 ± 0.19 ^c^	8.49 ± 0.48 ^4,c^	9.35 ± 0.42 ^a^	9.05 ± 0.32 ^b^	*F*_1,84_ = 87.8, *p* < 0.001	*F*_1,84_ = 2.96, ns	*F*_1,84_ = 4.36, *p* < 0.05
Wet weight (g)	0.933 ± 0.050	0.951 ± 0.082 ^4^	0.899 ± 0.064	0.944 ± 0.057	*F*_1,84_ = 2.21, ns	*F*_1,84_ = 5.35, *p* < 0.05	*F*_1,84_ = 0.89, ns
Dry weight (g)	0.686 ± 0.035	0.693 ± 0.055 ^4^	0.640 ± 0.050	0.672 ± 0.041	*F*_1,84_ = 12.0, *p* < 0.001	*F*_1,84_ = 4.04, *p* < 0.05	*F*_1,84_ = 1.71, ns
Volume (cm^3^)	0.596 ± 0.032	0.611 ± 0.058 ^4^	0.584 ± 0.042	0.618 ± 0.038	*F*_1,84_ = 0.06, ns	*F*_1,84_ = 6.73, *p* < 0.05	*F*_1,84_ = 0.99, ns
Density (g/cm^3^)	1.57 ± 0.02	1.56 ± 0.03 ^4^	1.54 ± 0.02	1.53 ± 0.02	*F*_1,84_ = 30.3, *p* < 0.001	*F*_1,84_ = 2.9, ns	*F*_1,84_ = 0.3, ns

Values are means ± SD; ^1^ Analyzed by two-way ANOVA; Fisher’s least significant difference post hoc test was performed for the parameter with a significant (*p* < 0.05) strain × diet interaction; Values within the row without a common superscript letter differ, *p* < 0.05; ns, *p* ≥ 0.05; ^2^ Length from the greater trochanter to the lateral condyle; ^3^ Midshaft mediolateral width; ^4^ *n* = 22.

## Supplementary Materials

The following are available online at http://www.mdpi.com/2072-6643/8/5/253/s1.
