# Supplementary material for: Moderately Low Magnesium Intake Impairs Growth of Lean Body Mass in Obese-Prone and Obese-Resistant Rats Fed a High-Energy Diet
Source: Nutrients. 2016 Apr 28;8(5):253. doi: 10.3390/nu8050253 (PMC4882666; doi:10.3390/nu8050253)
Supplement: Supplementary file 1 [file nutrients-08-00253-s001.docx]

Supplementary Material: Moderately Low Magnesium Intake Impairs Growth of Lean Body Mass in Obese-Prone and
Obese-Resistant Rats Fed a High-Energy Diet

Jesse Bertinato, Christopher Lavergne, Sophia Rahimi, Hiba Rachid, Nina A. Vu,
Louise J. Plouffe and Eleonora Swist

**Table S1.** Diet compositions.

| **Component** | **Diets** | |
| --- | --- | --- |
|  | **LMg** | **NMg** |
| Fixed ingredients (g/kg) ^1^ | 710 | 710 |
| Sucrose (g/kg) | 289.934 | 289.271 |
| Energy density (kcal/kg) ^2^ | 4395 (18.40) | 4393 (18.39) |
| CHO (% of energy) | 51 | 51 |
| Lipid (% of energy) | 33 | 33 |
| Protein (% of energy) | 16 | 16 |
| Mg oxide (g/kg) | 0.166 | 0.829 |
| Mg content (g/kg DW) ^3^ | 0.116 ± 0.001 * | 0.516 ± 0.007 |
| Ca content (g/kg DW) ^3^ | 5.54 ± 0.07 | 5.55 ± 0.09 |

^1^ Fixed ingredients (g/kg diet): cornstarch, 202.191; casein, 190; corn oil, 118; dyetrose, 75; anhydrous milkfat, 44.2; AIN-93G mineral mix without Mg (No.: 214505), 35; cellulose (microcrystalline),
30; AIN-93G vitamin mix (No.: 310025), 10; l-cystine, 3; choline bitartrate, 2.5; ethoxyquin, 0.009.
Ca, calcium; CHO, carbohydrate; DW, dry weight; Mg, magnesium; ^2^ Energy density in MJ/kg is shown in parentheses; ^3^ Analyzed content. Values are means ± SD, *n* = 5. * Different from the NMg diet by t-test, *p* < 0.001.


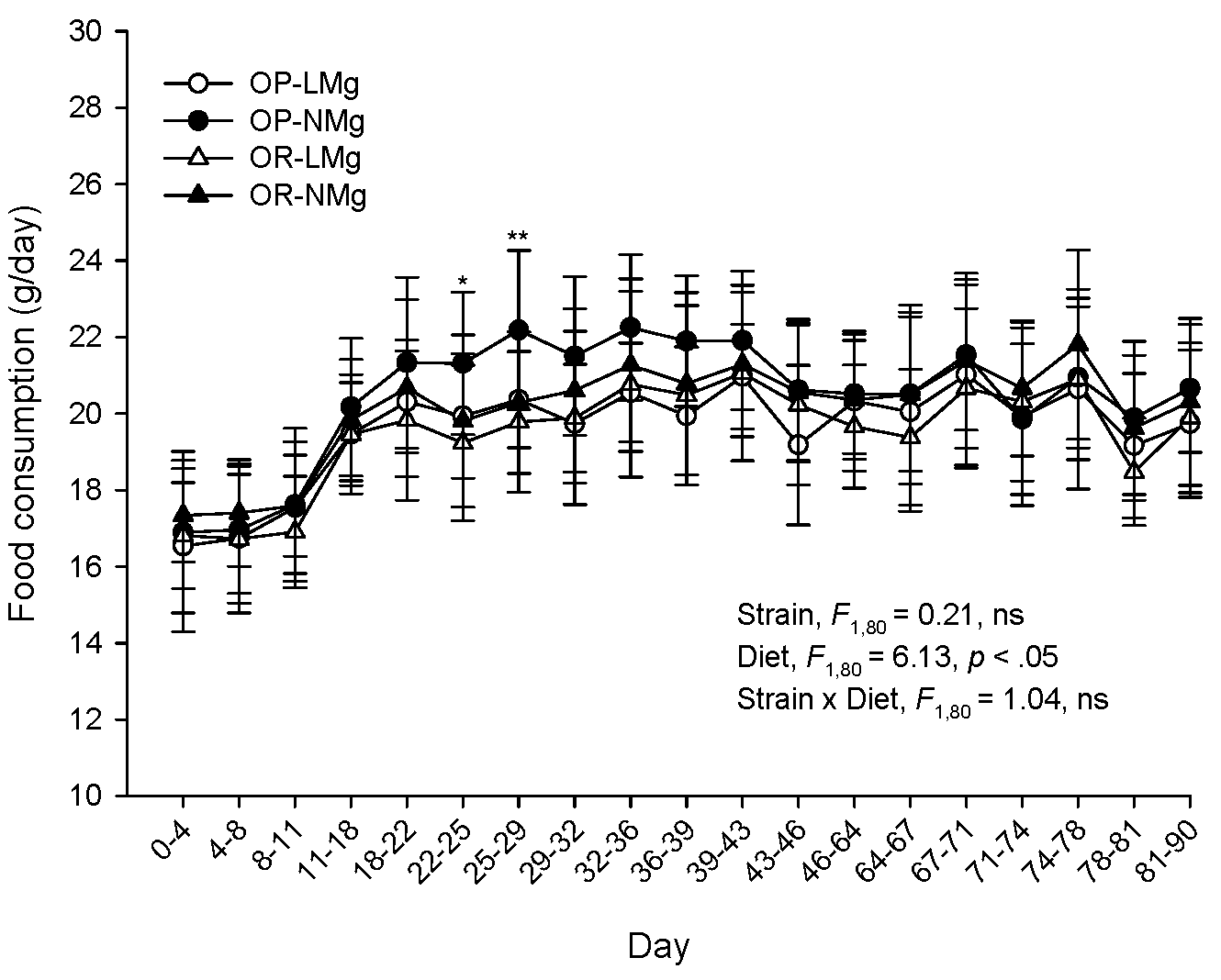


**Figure S1.** Food consumption of rats. Results are presented as means ± SD, *n* = 17–25. Results were analyzed by mixed-design ANOVA to determine effects and interactions of time, strain and diet.
A time × strain interaction (*p* < 0.05) was observed and univariate results are shown for effect of strain (*, *p* < 0.05; **, *p* < 0.01). ns, *p* ≥ 0.05.


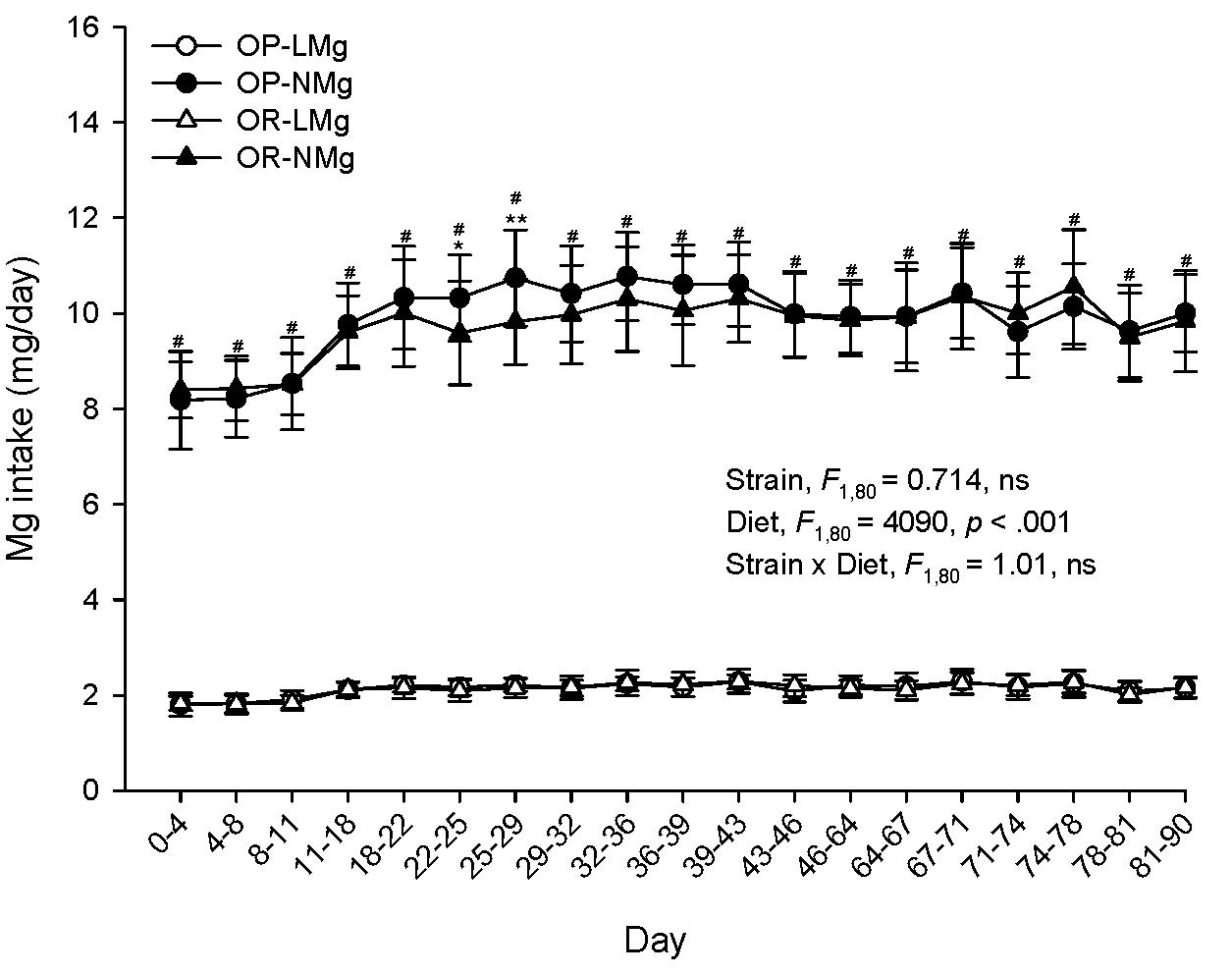


**Figure S2.** Mg intake of rats. Mg intake was calculated from food consumption and analyzed Mg content of the diets (based on wet weight). Results are presented as means ± SD, *n* = 17–25. Results were analyzed by mixed-design ANOVA to determine effects and interactions of time, strain and diet. Time × strain and time × diet interactions (*p* < 0.05) were observed and univariate results are shown for effects of strain (*, *p* < 0.05; **, *p* < 0.01) and diet (^#^, *p* < 0.001). ns, *p* ≥ 0.05. Mg, magnesium.
